# Practical strategies to enhance resident engagement in clinical quality improvement

**DOI:** 10.1186/s12909-022-03134-y

**Published:** 2022-02-14

**Authors:** James P. Koller, Kelly A. Cochran, Linda A. Headrick

**Affiliations:** 1 University of Missouri-Columbia School of Medicine, 101 S. Fairview Rd, Columbia, Missouri 65203 USA; 2 University of Missouri-Kansas City School of Pharmacy, Kansas City, Missouri 64108 USA

**Keywords:** Graduate medical education, Quality improvement, Practice based learning and improvement, Systems based practice

## Abstract

**Background:**

Engaging residents in meaningful quality improvement (QI) is difficult. Challenges include competing demands, didactics which lack connection to meaningful work, suboptimal experiential learning, unclear accountability, absence of timely and relevant data, and lack of faculty coaches and role models. Various strategies to address these challenges for engagement have been described, but not as a unified approach. This paper describes a bundle of practical strategies to address common challenges to resident engagement in QI, illustrated through the experience of one residency education program.

**Methods:**

62 categorical residents in the University of Missouri Internal Medicine residency participated in a longitudinal QI curriculum integrated into residency clinic assignments with dedicated QI work sessions and brief just-in-time didactics with mentorship from faculty coaches. Residents completed at least two PDSA (Plan-Do-Study-Act) cycles for their projects. The experience included clear expectations and tools for accountability. Project criteria included importance to patients, residents, and the institution. Residents had access to data related to their own practice. A pre-post survey asked residents to self-assess their level of interest and engagement in QI on a 5-point Likert scale, with 1 = least desired and 5 = most desired result. Data were analyzed by paired t-test.

**Results:**

All 62 residents participated in the program as members of ten QI teams. 40/62 residents completed both pre- and post-surveys. Items related to self-assessment of QI in clinical work all changed in the desired direction: likelihood of participation (3.7 to 4.1, *p* = 0.03), frequency of QI use (3.3 to 3.9, *p* = 0.001), and opinion about using QI in clinical work (3.9 to 4.0, *p* = 0.21). Resident assessment of QI priority in clinical work did not change.

**Conclusions:**

We implemented a practical strategies bundle to overcome common challenges to successfully engaging residents in clinical quality improvement. These strategies included QI work integrated into routine clinical assignments, just-in-time didactics, experiential learning with clear expectations and strategic project selection, timely and pertinent data from the residents’ own practice, and real-time faculty coaching.

**Supplementary Information:**

The online version contains supplementary material available at 10.1186/s12909-022-03134-y.

## Background

Engaging residents in quality improvement (QI) is difficult. Challenges include residents’ competing demands, didactics not connected with meaningful work, suboptimal experiential learning, lack of clear accountability, lack of timely and relevant data, and inadequate numbers of faculty to coach and role model [1–7]. (See Table 1).

**Table 1:** Bundle of Practical Strategies for Challenges to Engaging Residents in QI

Bundle of Practical Strategies for Challenges to Engaging Residents in QI	
Challenges	Strategies
Competing demands	• Longitudinal QI curriculum integrated into residency clinical assignments • QI work sessions scheduled in advance
Didactics not connected with meaningful work	• Brief, just-in-time didactics with immediate application in structured work sessions • Concepts illustrated with local examples of QI work
Suboptimal and incomplete experiential learning	• QI projects aligned with patient, resident and institutional priorities • Projects chosen by the residents in consultation with faculty and institutional clinical leaders • Key stakeholders identified and engaged by resident teams • Increasing QI team leadership as residents progress through the training program • QI interventions within the scope of influence of supportive clinical leadership
Lack of clear accountability	• Written expectations, assignments, roles and responsibilities • Resident teams’ self-assigned action steps at the end of each work session • Clear work products, e.g., Grand Rounds poster template for routine reporting
Lack of timely and relevant data	• Availability of data included in project selection criteria • Real-time data from the residents’ own practice, e.g., through a registry
Lack of faculty able to coach and role model	• Knowledgeable, interprofessional core faculty • Facilitator guide for easy faculty cross-coverage • Resident QI work sessions scheduled as part of routine faculty responsibilities • Real-time faculty coaching during QI work sessions

*Residents’ competing demands* include clinical assignments and scholarly research participation [5, 8–11]. Some programs struggle to find time to deliver their QI curriculum, and residents have to juggle all these activities. Residents complain that *quality improvement didactics are abstract* and disconnected to what they believe is most important in their work [4, 12, 13]. Classroom-based learning can be difficult to apply if it is not quickly linked to meaningful activities [14].

*Experiential learning in QI is necessary*; didactics cannot stand alone [4, 15, 16]. QI work is more likely to be successful if it is interesting to residents, impactful to patients, and important to the institution [13, 17]. Residents working on projects that align with institutional priorities are more likely to have access to institutional infrastructure such as QI experts, data analytics, and interprofessional involvement [1, 4, 8, 18, 19]. Unfortunately, residents often are unable to work through the full scope of an improvement effort [3, 7, 20, 21].

*Unclear expectations of residents and lack of accountability* can also limit residents’ QI engagement. Without clear deliverables and deadlines, project work is easy to put off until the last minute or not complete at all. This may be especially true when residents are expected to do project work on their own time [1].

Successful QI work depends on *data that is timely and relevant* to practice. Many institutions have limited infrastructure for data gathering and analysis. Those resources rarely are available to residents, especially for resident-generated projects [4, 6, 8, 19, 22, 23]. Manual medical record reviews can be helpful, but the burden of data collection often makes that approach unsustainable.

Many academic health centers lack sufficient numbers of *faculty prepared to teach and coach QI* [2, 4, 15, 21, 24]. Faculty cite inadequate time to work with resident QI teams [4, 5]. In addition, faculty vary in their assessment of the relative importance of QI [6, 25].

The literature proposes a variety of methods to address these challenges, but not as a unified approach. This paper describes a bundle of practical strategies to address these challenges to resident engagement: incorporating QI into the residents’ clinical assignments, providing brief just-in-time didactics, constructing meaningful experiential learning, setting clear expectations for work products, using timely data from the residents’ own clinical practices, and involving faculty equipped and available to coach and role model. (See Table 1.) We illustrate their unified application through the experience of one residency program.

## Methods

### Setting and participants

During the 2017–18 academic year, there were 62 residents who were in their first, second, or third post-medical school graduate year at the University of Missouri Internal Medicine residency. The residency was organized in an X + Y schedule, specifically 4 + 1, meaning that residents spent 4 weeks on an assigned rotation outside of the continuity clinic, then 1 week full-time in their primary care continuity practice [26, 27]. Continuity clinic is a resident general internal medicine primary care practice. Residents follow their own primary care patients for 3 years. These continuity clinics took place either at the University of Missouri General Internal Medicine Clinic (MU Health) or at the Harry S. Truman VA Internal Medicine Clinics (HSTVA). The PGY 1–3 residents with the same clinic week assignment (*n* = 11–13) made up a cohort; there were a total of five cohorts. Residents in each cohort were further divided into two QI teams.

### Intervention

Three faculty members developed and delivered the QI curriculum: two internal medicine faculty members [one an associate program director (APD) and MU Health clinic medical director, the second a QI expert] and a pharmacy faculty member. Table 2 lists the curriculum’s learning objectives, built on the Model for Improvement QI theoretical framework [28]. We applied the following bundle of strategies in an attempt to maximize resident engagement. Specifically, we addressed competing demands, instituted just-in-time didactics, incorporated experiential learning, established accountability, addressed data issues, and provided faculty coaching.

**Table 2:** Resident QI Curriculum Learning Objectives

Resident QI Curriculum Learning Objectives	
1. Describe criteria for choosing a QI project	
2. Conduct a first QI team meeting using the 7-step meeting process	
3. Identify elements that contribute to successful teamwork	
4. Draft a SMART aim	
5. For the team’s QI project, answer the first two questions in the Model for Improvement (What are we trying to accomplish? How will we know that a change is an improvement?)	
6. Draft operational definition(s) for key project measure(s)	
7. Describe how process knowledge contributes to improvement	
8. Use a fishbone diagram to explore the gap between current and desired results	
9. Use Rogers’ characteristics of successful innovation to plan a test of change	
10. Use an effort/yield 2 × 2 table to analyze ideas about changes to test	
11. Plan a PDSA cycle, something that can be done quickly, with results by next session. Keep as small as possible.	
12. Draft an IRB QI application	
13. Review results of first PDSA cycle	
14. Complete “Act” of PDSA by identifying supports and barriers in PDSA cycle #1 to inform the PDSA cycle #2	
15. Plan PDSA cycle #2: Sustain/spread the intervention tested in the first PDSA cycle? Try something new?	
16. Describe the utility of an annotated run chart	
17. Review updated QI poster, with results of first two PDSA cycles	
18. Identify lessons so far	
19. Plan strategies for project completion by May 2018	
20. Compare the analytic power of data displayed in a table with data displayed in a run chart	
21. Use run chart analysis rules to distinguish significant change from random variation	
22. Review updated QI poster, with results of first three PDSA cycles	
23. Reflect on PDSA cycles so far to identify lessons about making and sustaining change	
24. Use a Force Field diagram to identify drivers and barriers to desired change. Brainstorm ways to strengthen drivers and weaken barriers.	
25. Name 2–3 “take homes” for your next QI project; use those to inform the “lessons learned” part of the final poster	

Addressing competing demands: Each cohort of residents formed two QI teams of 3–8 residents. The APD scheduled a one-hour QI work session along with other required activities during each ambulatory block week. Each session reflected specific learning objectives, with a faculty presentation (“theory burst”) of QI content (< 15 min) followed by resident QI teamwork.

Just-in-time didactics: One week before the teaching session, the APD emailed the topic to the residents and gave them a pre-session reading assignment of only a few pages. Each face-to-face session began with a 20-min theory burst covering essential QI knowledge and skills, illustrated with local examples. The rest of the time was devoted to resident QI team meetings with work products requiring the residents to apply what they had just learned. Educational materials such as worksheets and agenda templates for resident team meetings provided prompts for key tasks, encouraged explicit team roles, and served as a record of assignments to be accomplished before the next session.

Experiential learning: Resident QI teamwork focused on a project chosen by the residents in consultation with the faculty and institutional leaders. Recommended criteria for project selection included 1) important to patients, residents, and the institution; and 2) data available over time. Eight teams had projects based at their clinic site within MU Health or HSTVA; two teams had projects based in the inpatient internal medicine unit at MU Health or HSTVA. Senior residents (PGY 2 and 3) assumed leadership roles within their QI teams. Resident teams engaged stakeholders from the clinical learning environment (including nurses, attending physicians, administrators, data analysts, and clinical pharmacists) in various aspects of the project.

Accountability: The pre-session email from the APD included work session product expectations. Teams tracked their specific aim, measures, PDSA cycles and outcomes as the project progressed using a poster template (Appendix 1). Residents were accountable for sharing their updated poster template with lead faculty prior to the start of each QI session for feedback. At the end of each QI work session, the teams reported out, sharing their progress and self-assigned action steps. Each resident team was required to complete at least two PDSA cycles. At the end of the academic year, residents presented their finished poster at Internal Medicine Grand Rounds and were encouraged to submit abstracts to regional and national meetings.

Timely and relevant data: Teams utilized data from their own clinical practice. The four teams with projects at the MU Health continuity practice accessed data through Cerner’s Healthy Intent® health registry, which drew real time patient data from the electronic health record. Residents easily accessed the health registry data for their own patient panel, their cohort and the general internal medicine clinic overall. To maintain patient privacy, data for the cohort or clinic overall was not identified by patient and presented only as group data. Electronic data was not available for four outpatient-based projects at the VA and one inpatient project at MU Health; residents on those teams manually collected data through medical record reviews and paper surveys.

Faculty coaching and role modeling: Faculty participation in the resident QI work sessions was scheduled as part of routine faculty responsibilities. At least one of the core faculty members was present at each QI work session; often all three attended. Since each session repeated five times (once for each resident cohort), the faculty prepared a facilitator guide (Appendix 2) to ensure consistency from one week to the next. Faculty provided feedback to the resident QI teams during the work session. Faculty modeled QI by using a “minute paper” to collect resident feedback at the end of each session; thus improvements could be made from one week to the next [29].

### Data collection and analysis

We developed a six-question, pre-post survey which we administered prior to the start of the QI curriculum and at the end of the academic year. The instrument asked residents to self-assess their level of interest and engagement in QI on a 5-point Likert scale, with 1 = least desired and 5 = most desired result. Survey results were analyzed by paired t-test.

A faculty QI expert evaluated each final Grand Rounds poster for evidence of QI learning consistent with key learning goals: 1) Aim was specific, measurable, attainable, and reasonable and has a time frame (SMART) [30]. 2) Project measures were related to the overall aim. 3) Interventions were based on process knowledge. 4) At least two PDSA cycles were completed. 5) Data were graphically displayed over time.

## Results

All 62 residents participated in the QI education program as members of ten teams. Each team completed a QI project and submitted a poster for the Department of Medicine’s annual QI Grand Rounds. Examples of projects included improvements in hepatitis C screening, LDL monitoring in diabetes, and colon cancer screening for primary care patients. Members of the department QI committee rated resident posters highly compared to fellow and attending submissions, disproportionately selecting resident QI projects for four of the five oral presentations at Grand Rounds. Three resident teams submitted abstracts for competitive review at regional or national meetings; two were accepted.

**Fig. 1:**
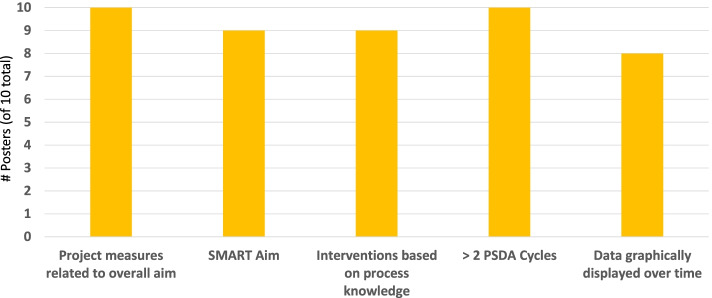
Resident QI Learning Documented in Posters. Number of resident team QI posters demonstrating achievement of key QI education program learning goals

Final team posters demonstrated achievement of key learning goals (Fig. 1). All ten described at least two completed PDSA cycles with measures related to the overall aim, nine had a SMART aim, nine described interventions based on process knowledge, and eight graphically displayed data over time.

**Fig. 2:**
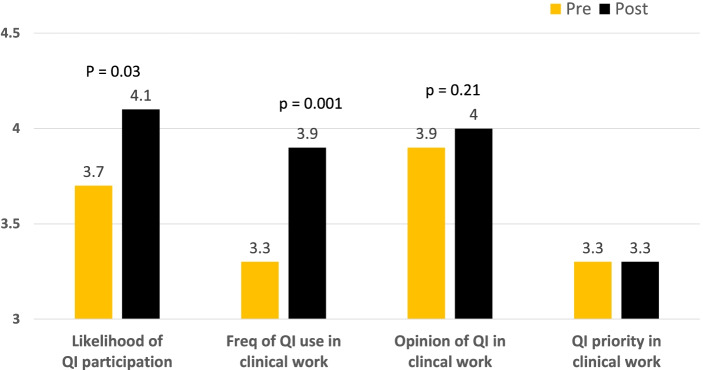
Resident QI Engagement. Responses to pre/post surveys, *n* = 40/62 (64.5%). Key to scales. Likelihood: 1 = Extremely Unlikely, 2 = Unlikely, 3 = Neutral, 4 = Likely, 5 = Extremely Likely. Frequency: 1 = Never, 2 = Rarely, 3 = Occasionally, 4 = Frequently, 5 = Always. Opinion: 1 = Skeptical, 2 = Not interested but acknowledge it has to be done, 3 = Indifferent, 4 = Interested, 5 = Enthusiastic. Priority: 1 = Not a priority, 2 = Low, 3 = Medium, 4 = High, 5 = Essential

Matched pre- and post-survey were available from 40/62 (64.5%) residents. Surveys were matched by number to preserve anonymity of the respondents. Fig. 2 shows that all the items related to self-assessment of QI engagement changed in the desired direction, with likelihood of QI participation (*p* = 0.03) and anticipated frequency of QI use in clinical work (*p* = 0.001) reaching statistical significance. The residents’ assessment of QI as a priority in clinical work did not change and the opinion of QI in clinical work slightly improved.

In the written feedback at the end of session, residents rated the sessions highly and offered helpful suggestions for improvement. For instance, we adjusted the time of the work session to the hour immediately after morning report, clustering the residents’ scheduled obligations.

## Discussion

In our QI curriculum, we applied a bundle of practical strategies to address common challenges to engaging residents in meaningful QI activities. The residents accomplished key learning goals and improved their QI engagement.

### Addressing competing demands

QI experiences are limited and learning objectives are difficult to achieve when competing demands prevent learners from accomplishing desired tasks [1, 4, 8]. When residents are unable to see the results of their QI work, their engagement is threatened [9, 31].

Facilitated by the 4 + 1 framework, our QI didactics and project work took place during dedicated time away from research or clinical responsibilities. The sessions occurred during daytime work hours, facilitating connections with important stakeholders. With advance notice, faculty were able to adjust their calendars to attend these sessions. Others have reported the value of explicitly scheduling resident QI learning, whether as part of the residents’ core curriculum time [32, 33], as part of an x + y model [18] or in specific clinical assignments [12, 13, 21, 34].

We structured QI work sessions so that resident teams accomplished specific parts of the QI project in each session, clearly connecting project-related activities to learning objectives for the day. Faculty coached the teams during these sessions, helping the residents complete the work more efficiently. In end-of-curriculum feedback, residents reported that they were successful in completing the QI work in the dedicated time.

### Just-in-time didactics

To facilitate the transfer of classroom-based learning to professional practice, we provided just-in-time didactics coupled with opportunities for the residents to immediately apply key concepts to their QI project [14]. Others have taken a similar approach [18, 32]. The literature cites the effectiveness of the flipped classroom for successful adult learning, but pre-session assignments compete with other demands for resident time [18, 35, 36]. We made pre-session work assignments very brief, focusing on the essentials the residents needed to support the experiential aspects of their learning [37].

### Experiential learning

Goldman et al. described experiential learning as “constructing knowledge and meaning from real-life experience” and, in health professions education, “learning that is triggered by authentic practice-based experience.” [4] We sought to construct the residents’ experiential learning in a way that aligned with patient, resident and institutional priorities, while allowing residents to make the final choice about their QI project focus. In combining these strategies, we attempted to tap into the power of *bidirectional alignment*; that is, allowing QI priorities to originate from the point-of-view of learners as well as that of leaders. Johl and Grigsby argued that this approach informs institutional priorities with learners’ fresh perspectives, fewer preconceived notions and a learning mindset [11]. We also sought to increase residents’ engagement by making it possible for them to focus their work on issues that affected their and their patients’ own lives [13]. Institutions and GME need to work together to pick the projects in which the residents participate and ensure they are not just bystanders [2, 6, 7, 19].

In our residents’ experiential learning, we emphasized the importance of engaging other stakeholders; this was a focus of much of the faculty’s coaching. While we invited clinical team members and stakeholders to specific QI work sessions, they were not incorporated as members of the residents’ QI teams. Given the importance of interprofessional collaboration to the success of clinical improvement, we suspect that more consistent involvement of interprofessional collaborators would have enhanced the success of the resident QI projects as well as fostered meaningful learning around the contributions of healthcare partners in improvement work.

In our program, one or more PGY2 or PGY3 residents led each project. In a national survey of graduating family medicine residents, Lichkus et al. found that residents reporting experience in QI team leadership roles were almost twice as likely to report feeling extremely prepared (versus moderately prepared) to lead QI projects [38]. Clarke et al. argued that neurosurgery resident QI interest and participation was reinforced by placing them in project leadership roles [9].

The longitudinal nature of our residents’ experience made it possible for the faculty to require that each resident team complete at least two PDSA cycles, supporting learning through both success and failure in tests of change. Co noted that a longitudinal approach to experiential learning allows learners to develop projects that require more time for planning and execution [2]. This is critical, as unfinished projects by definition limit the residents’ ability to experience the entire QI cycle [7] and may contribute to resident frustration, disengagement and resistance to future QI efforts [4].

### Accountability

Unclear expectations for residents’ QI work and lack of accountability can limit the success of their learning. We addressed this with the following: 1) written expectations, assignments, roles and responsibilities; 2) asking the residents to report their self-assigned action steps at the end of each work session; and 3) requiring clear work products, including a poster template that the residents updated and submitted to the faculty before each work session and finalized at the end for submission to Internal Medicine QI Grand Rounds. Many authors report the value of setting expectations for routine reporting and feedback [31, 39–42]. Many programs require a final product, such as a poster or oral presentation [10, 19, 33, 41, 43]. In the previously cited survey, Lichkus et all found that completion of a written summary for academic presentation in combination with QI team leadership had the strongest association with perceived preparedness for QI work [38].

### Timely and relevant data

Recognizing that successful quality improvement requires timely and relevant data, the faculty included data availability as part of the recommended criteria for project selection. The residents with projects that accessed data from the health registry were able to analyze pertinent patient-based data at any time and at multiple levels: individual physician patient panel, by resident cohort, and for the general internal medicine practice as a whole. Those QI teams could see the results of their interventions rapidly, from one PDSA cycle to the next.

Projects that needed data outside the registry (either due to the project aim or due to the care site, such as inpatient) ultimately relied on manual data collection or paper surveys. Despite the best intentions of institutional data managers, other priorities prevented them from responding to data requests in time to meet resident project needs. Predictably, manual data collection was possible for only small numbers of patients, limiting the impact, sustainability and spread of the work. In their large, health system-wide initiative, Vidyarthi et al. reported that resident QI activities that used data collection techniques designed specifically for that project had difficulty achieving their goals [17]. Ultimately, they too encouraged residents to align their projects with efforts for which data were already being collected. Selecting projects with already-available data is a reasonable and practical solution, but it limits the possibilities for QI work. Tess et al., called for institutional mechanisms to expand data access at the level of both the trainee and the residency program [6].

### Faculty coaching and role modeling

Multiple studies cite access to QI expertise and knowledgeable project coaching as major factors in the success of QI learning [3, 4, 8, 24, 38]. Goldman et al. described the following as important elements of project coaching: guidance around QI methods and principles, understanding of organizational contexts, access to hospital data, clinical or QI experience, and multiprofessional perspectives [4].

At our institution, three QI-savvy faculty formed an interprofessional team to teach QI sessions and provide project coaching for the residents. A facilitator guide made it easy for us to cover for each other, so that all did not have to be present at every session. We found it helpful for a consistent faculty team to work with the residents throughout their projects, providing real-time feedback. As in our model, other QI education programs have found ways to schedule faculty QI teaching and coaching as part of their routine work [34, 41, 42]. Support of faculty time demonstrates a commitment to QI that makes it easier to establish the role clarity and performance expectations (such as scheduling routine meetings) that contribute to successful project coaching [4, 40, 44].

We role modeled QI in our work as educators by soliciting and responding to resident feedback, even from session to session. We highlighted local QI efforts and connected the residents to other faculty QI role models. We believed that to do otherwise would undercut our message of the importance of QI in health care.

### Limitations

We are confident that our approach will work in any X + Y curriculum but are uncertain of its application to a traditional once-weekly continuity clinic paradigm. We developed our own survey instrument, since at the time there was no previously validated tool to assess residents’ sense of prioritization and participation in QI as part of their clinical work. In addition, it would have been interesting to explore with the residents why their priority of QI in clinical work did not change when other responses changed in the desired direction. This may be a topic worth exploring in future research.

## Conclusion

In this paper, we identify and address common challenges to teaching residents about QI, using the University of Missouri experience as an illustration. Our practical strategies resulted in improved resident engagement. These included addressing competing demands by integrating resident QI work into routine work assignments, delivering just-in-time didactics, and implementing meaningful experiential learning. Important to experiential learning were clear communication of what we expected from the residents, strategic project selection, and resident ownership of projects. Timely and pertinent data from a patient registry and real time faculty coaching and role modeling contributed to completion of resident QI work. Future work would be to test this bundle of strategies in other GME programs looking to start or improve their QI education programs.

## Supplementary Information

**Additional file 1.**

**Additional file 2.**

## Data Availability

All data generated and analyzed during this study are included in this published article.

## Abbreviations

APD: Associate Program Director
GME: Graduate Medical Education
HSTV: Harry S Truman Veterans Hospital
MU: University of Missouri
PDSA: Plan-do-study-act
PGY: Post graduate year
QI: Quality Improvement
SMART: Specific, Measurable, Attainable, Relevant, Timely
VA: Veterans Administration
