# Supplementary material for: Practical strategies to enhance resident engagement in clinical quality improvement
Source: BMC Med Educ. 2022 Feb 14;22:96. doi: 10.1186/s12909-022-03134-y (PMC8842865; doi:10.1186/s12909-022-03134-y)
Supplement: Supplementary file 1 — Additional file 1. [file 12909_2022_3134_MOESM1_ESM.pdf]

Health system logo

TITLE

**Start Date:**<br>**Report Date:**	**Executive Sponsor(s):**	**Team Leader:**<br>**Team Members:**

Background:

Overall Aim Statement	PDSA 1	PDSA 2	PDSA 3	PDSA 4
	**Specific Aim:**	**Specific Aim:**	**Specific Aim:**	**Specific Aim:**
	**Specific Measure:**	**Specific Measure:**	**Specific Measure:**	**Specific Measure:**
**Overall Specific Measures**	**Intervention**	**Intervention**	**Intervention**	**Intervention**

Graphics / Data for Overall Results	Recommendations for the Future

University of Missouri, 2022
