# Supplementary material for: Practical strategies to enhance resident engagement in clinical quality improvement
Source: BMC Med Educ. 2022 Feb 14;22:96. doi: 10.1186/s12909-022-03134-y (PMC8842865; doi:10.1186/s12909-022-03134-y)
Supplement: Supplementary file 2 — Additional file 2. [file 12909_2022_3134_MOESM2_ESM.docx]

*Facilitator Guide Template*

**Session 1: Title**

**Division**

**Faculty**

**Prerequisites/Readings Prior to Session 1:**

- Include specific readings with corresponding page numbers and links or files

**Homework for Session 2:**

- Include specific expectations for any work to be done between sessions

**Purpose:** describe intention of the session

**Learning Objectives:**

- Identify characteristics and habits of an effective team
- Practice using a timed agenda
- Draft a SMART aim

**Resource/Supplies:** (number needed; specific description of items; where to obtain; where to return; specific layout of supplies/room, including pictures or diagrams if needed)

| **Start Time** | **Allotted Time** | **Activity Description** | **Discussion Points/Pearls** |
| --- | --- | --- | --- |
| 10:00a | 10min |  | -Include room set up  -Any specific reminders for facilitator to articulate |
| 1040a | 5 min |  |  |
| 1045a | 10 min |  |  |
| 1055a | 20 min |  |  |
| 11:15a | 15min | **Wrap Up:**  **Any Questions?**  **Homework for Session 2** |  |
|  |  | **Minute Papers completed** |  |

**Detailed Activity Description:**

**Discussion Questions/Prompts:**
